# Random Forest Analysis of Untargeted Metabolomics Data Suggests Increased Use of Omega Fatty Acid Oxidation Pathway in Drosophila Melanogaster Larvae Fed a Medium Chain Fatty Acid Rich High-Fat Diet

**DOI:** 10.3390/metabo9010005

**Published:** 2018-12-31

**Authors:** Vishal H. Oza, Joseph K. Aicher, Laura K. Reed

**Affiliations:** Department of Biological Sciences, University of Alabama, Tuscaloosa, AL 35487, USA; jkaicher@crimson.ua.edu (J.K.A.); lreed1@ua.edu (L.K.R.)

**Keywords:** untargeted metabolomics, Drosophila melanogaster, random forest, Gaussian graphical models

## Abstract

Obesity is a complex disease, shaped by both genetic and environmental factors such as diet. In this study, we use untargeted metabolomics and Drosophila melanogaster to model how diet and genotype shape the metabolome of obese phenotypes. We used 16 distinct outbred genotypes of Drosophila larvae raised on normal (ND) and high-fat (HFD) diets, to produce three distinct phenotypic classes; genotypes that stored more triglycerides on a ND relative to the HFD, genotypes that stored more triglycerides on a HFD relative to ND, and genotypes that showed no change in triglyceride storage on either of the two diets. Using untargeted metabolomics we characterized 350 metabolites: 270 with definitive chemical IDs and 80 that were chemically unidentified. Using random forests, we determined metabolites that were important in discriminating between the HFD and ND larvae as well as between the triglyceride phenotypic classes. We found that flies fed on a HFD showed evidence of an increased use of omega fatty acid oxidation pathway, an alternative to the more commonly used beta fatty acid oxidation pathway. Additionally, we observed no correlation between the triglyceride storage phenotype and free fatty acid levels (laurate, caprate, caprylate, caproate), indicating that the distinct metabolic profile of fatty acids in high-fat diet fed Drosophila larvae does not propagate into triglyceride storage differences. However, dipeptides did show moderate differences between the phenotypic classes. We fit Gaussian graphical models (GGMs) of the metabolic profiles for HFD and ND flies to characterize changes in metabolic network structure between the two diets, finding the HFD to have a greater number of edges indicating that metabolome varies more across samples on a HFD. Taken together, these results show that, in the context of obesity, metabolomic profiles under distinct dietary conditions may not be reliable predictors of phenotypic outcomes in a genetically diverse population.

## 1. Introduction

A high-fat diet has been associated with many metabolic disease states such as diabetes, obesity, cardiovascular diseases, and metabolic syndrome [1,2,3,4,5,6,7]. Drosophila melanogaster has emerged as one of the important model organisms for evaluating the molecular and genetic mechanisms of these diseases [8,9,10,11,12,13,14]. Despite the extensive use of Drosophila in understanding human disease pathways the effects of diet on metabolic phenotypes requires further elucidation. Previous studies in Drosophila [15,16,17] have established diet as one of the important environmental factors contributing to metabolic phenotypes in a genetically variable population. Here we have employed an untargeted metabolomics approach to characterize the differences in the global metabolic profile of Drosophila melanogaster in different environmental states (diet) and in distinct phenotypic responses to diet for triglyceride storage (reaction norms).

Untargeted metabolomics takes a high-resolution snapshot of the complete metabolome of an organism at any given instant [18], thus, due to its high throughput, and sensitivity, untargeted metabolomics has potential to find novel physiological patterns across diverse samples. Also, the number of previously uncharacterized metabolites is substantial and the relationships between these unknown metabolites go beyond those in canonical metabolic pathways [19]. Thus, untargeted metabolomics on a global scale can provide a “phenotypic readout” to identify altered biochemical pathways in diseases and help to elucidate the molecular mechanisms of novel biological processes [18]. 

In this study, Drosophila larvae from 16 wildtype genotypes, representing three environmental interaction (reaction norm) phenotypic groups for triglyceride storage, were fed on normal (ND) and high-fat diet (HFD). The reaction norm phenotypes were larvae that stored more triglyceride on a high-fat diet than on a normal diet (*N < HF*), larvae that stored more triglyceride on normal diet than a high-fat diet (*N > HF*) and, larvae that displayed no change (0) in triglyceride storage on either of the two diets. The larvae samples were then analyzed using liquid chromatography/mass spectrometry (LC/MS, LC/MS2) and gas chromatography/mass spectrometry (GC/MS) to obtain relative metabolite abundance data. From these data, we focus on two main questions: (1) Do the metabolic profiles differ between diets? and (2) Do the genetically variable organisms with similar diet-specific phenotypes (*N > HF, 0,* and *N < HF*) have the same metabolic profile? The data were analyzed using random forests, a machine learning method widely used to identify variables that best classify the data into different groups [20]. The advantages of the random forest algorithm are: (1) it avoids overfitting the data; (2) it gives an estimate of the important variables in the data with respect to the random forest classifier; and (3) it gives an inherent mean prediction error estimate for each training sample by evaluating predictions on those trees that did not include that sample and thus does not require a validation set for cross-validation; (4) relatively tolerant towards outliers [21]. Compared to other classification methods, random forests has been shown to perform better on metabolomics data for phenotypic classification and biomarker selection [22]. To further understand the interactions among metabolites as a metabolic network across dietary conditions, we also modeled the metabolic profiles as Gaussian graphical models (GGMs). GGMs represent conditional dependences between variables as edges between nodes in a network; any two variables X and Y in a GGM have an edge between them if and only if X and Y are statistically dependent given all remaining variables [23,24,25]. GGMs can learn dependencies between variables even in high-dimensional settings as found in metabolomics and other -omics studies [26].

## 2. Results

### 2.1. LC/MS and GC/MS Analysis

The global untargeted metabolic profile of Drosophila across diet and triglyceride phenotypes revealed a total of three hundred and fifty metabolites (Supplementary Table S1). Two hundred and seventy have definitive chemical IDs, and eighty metabolites were unknown. Two hundred and thirty-eight (Supplementary Table S1) map to PubChem [27], Kyoto Encyclopedia of Genes and Genomes (KEGG) [28,29] and/or the Human Metabolome Database (HMDB) [30].

### 2.2. Random Forest Analysis for Diet

**Figure 1:**
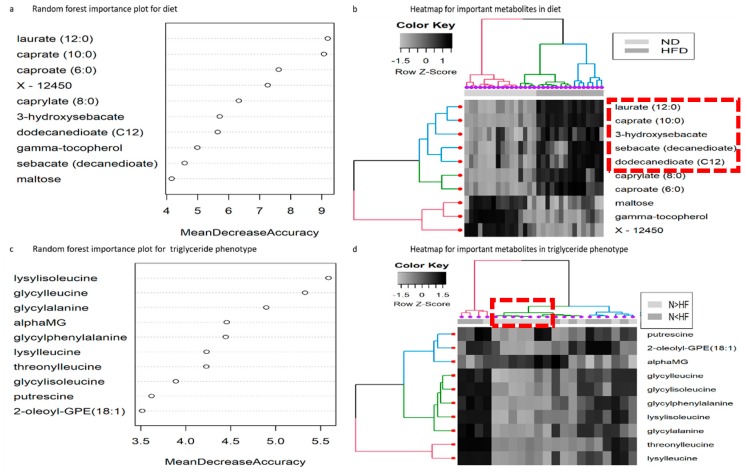
(**a**) Metabolite importance (top 10) calculated by Mean Decrease Accuracy for classification between a high-fat diet and normal diet. ntree = 1000, mtry = 18, OOB error rate = 3.23%, class error rate: high-fat diet = 0.0, normal diet = 0.06. (**b**) Heat map showing hierarchical clustering using Ward’s algorithm for the top 10 important metabolites identified by random forest for high-fat and normal diet. The clustering is done by sample (dark grey = high-fat, light grey=normal, along *x*-axis) as well as by metabolites (along *y*-axis). The red, green, and blue colors indicate the three distinct clusters identified. The red box indicates metabolites potentially involved in omega-fatty acid oxidation (**c**) Metabolite importance (top 10) calculated by Mean Decrease Accuracy for classification between reaction norm phenotypes (*N > HF*) and (*N < HF*). ntree = 1000, mtry = 18, OOB error rate = 23.81%, class error rate: *N > HF* = 0.33, *N < HF* = 0.16. (**d**) Heat map showing hierarchical clustering using Ward’s algorithm for the top 10 important metabolites identified by random forest for reaction norm phenotypes: The clustering is done by sample (dark grey = *N < HF*, light grey = *N > HF*, along *x*-axis) as well as by metabolites (along *y*-axis). The red, green, and blue colors indicate the three distinct clusters identified. The red box indicates three genotypes corresponding to *N < HF* phenotype exhibiting the same concentration profile for dipeptides irrespective of being on high-fat or normal diet.

In order to identify metabolites as important differentiators between two diets (normal and high-fat), we preformed random forest analysis. Some of the key differentiators within the top 15 were medium chain fatty acids, caproate (C6), caprylate (C8), caprate (C10), and laurate (C12), along with dicarboxylic fatty acids: decandionate (sebacate C10) and dodecandionate (C12), and monohydroxy fatty acids: 3-hydroxysebacate, 3-hydroxydecanoate, and 9-hydroxy-10,12-octadecadienoic acid + 13-hydroxy-9,11-octadecadienoic acid (9-HODE+13-HODE) (Top 10 important metabolites are shown in Figure 1a, and rest are in Supplemental Table S2). Several unknowns, labeled X-12450, X-19246, X-3452, X-17008, and X-18787 were also identified as important differentiators in our model (Supplemental Table S2). Hierarchical clustering showed two distinct clusters (Figure 1b) based on their abundance in HFD larvae and ND larvae. The medium chain fatty acids, dicarboxylic fatty acids, and certain mono-hydroxy fatty acids, have higher abundance in HFD larvae. In the case of ND larvae, unknown metabolite X-12450 along with maltose and gamma-tocopherol formed a cluster and were in higher abundance relative to HFD (Figure 1b). It should be noted that a number of the metabolites with significant differences between the two diets in ANOVA analyses (Supplemental Table S3), were not among the most important metabolites by random forest analysis (Supplemental Table S2). Of the 34 metabolites significant for diet differences at the *p* < 0.05 level in the ANOVA, 14 were not represented among the 34 most important metabolites. These additional metabolites were well distributed across metabolic pathways, showing no particular enrichment for any one aspect of metabolism. 

### 2.3. Random Forest Analysis for Reaction Norm Phenotypes

The random forest analysis for the two reaction norm phenotypes *N > HF* and *N < HF* show the most important metabolites in the phenotype contrast to have an “importance” that is about half that seen in the diet differentiating metabolites (Figure 1c, Supplemental Table S2), indicating that there is much lower signal in the phenotype contrast. Seven out of the top ten key differentiators were dipeptides (glycylleucine, glycylisoleucine, glycylphenylalanine, glycyclalanine, lysylisoleucine, lysylleucine, and threonylleucine; Supplemental Table S2). The dipeptides produced a strong clustering effect for a subset of samples from the same genotypes irrespective of diet, but overall, the ability to differentiate the reaction norm groups by clustering was weak (Figure 1d). However, it cannot be determined from this data whether the higher abundance of dipeptides in the larvae storing less triglyceride on a high-fat diet is because of elevated protein catabolism (perhaps due to oxidative stress) or because of enzyme-based synthesis mechanism to produce elevated levels of biologically active di-peptides as seen in bacteria (perhaps due to the gut microbiome) [31]. Similarly, to the distinction between the most important and most significant metabolites for diet above, there was little concordance between the most important and the most significant metabolites in the contrast of phenotypes. Of the 49 metabolites significant at the 0.05 level for the phenotype contrast (Supplementary Table S3), 20 were not among the 49 most important metabolites (Supplementary Table S2). These significant metabolites included 12 members of amino acid metabolism pathways, 12 lysolipids, as well as additional dipeptides. When we considered the triglyceride storage independent of the diet or reaction norm phenotype, we found eight lysolipids to be negatively correlated with triglyceride levels, and an additional 11 compounds from diverse chemical classes (Supplemental Table S3).

### 2.4. Gaussian Graphical Model of the Metabolic Pathways in Diet

**Figure 2:**
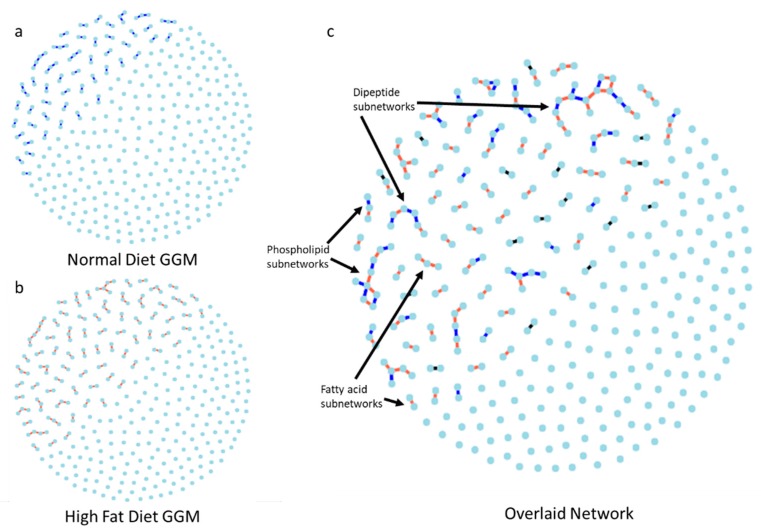
GGM showing relationships between metabolites in larvae fed with (**a**) Normal diet (blue) and (**b**) High-Fat diet (red) (**c**) The normal and high-fat diet networks are overlaid showing the unique normal diet model edges in blue, unique high-fat diet edges in red, and the common edges between the two in black (Interactive network visualization can be found in Supplemental Files: Figure_S2a_normal_ggm.html, Figure_S2b_highfat_ggm.html, and Figure_S2c_combined_ggm.html).

GGM constructed for ND was sparse with 57 edges (Figure 2a and Supplemental Table S4) compared to the HFD GGM that had 93 edges (Figure 2b and Supplemental Table S4). The two network models, when overlaid (Figure 2c), showed very few common network structures. Interestingly, the phospholipid and dipeptide metabolites formed subnetworks with edges coming from both diet networks, indicating variation across samples within a diet did not influence these pathways as a whole, but instead affected specific sub-steps. The dipeptide metabolites were also identified as important metabolites that differentiated between the two reaction norm phenotypes (*N > HF* and *N < HF*) by the random forest algorithm. Thus the variation among samples captured as edges in these pathways in Figure 2, may be linked to phenotypic effects. Four out of five metabolites involved in omega fatty-acid oxidation, identified as important differentiators by random forests, formed edges in the HFD network but not in ND (Figure 2c). Indicating that when studying the metabolome, it is important to study the change in network topology through the co-varying metabolites and not just the abundance change between two conditions. 

Understanding the network topology allows us to identify the underlying pathways that led to the difference in conditions, while differences in abundance will only identify metabolites showing the most drastic individual changes. To understand how the network topology changes, we calculated the edge symmetric difference (esd) between the HFD and ND networks to be 0.786 indicating that the two networks differed substantially in terms of the network topology. As a comparison, Landry et al. [32] captured traffic on the internet four months apart and calculated an esd of 0.25, indicating that 75% of the IP addresses pairs that were exchanging data at the first time point were also exchanging data at the second time point. 

**Figure 3:**
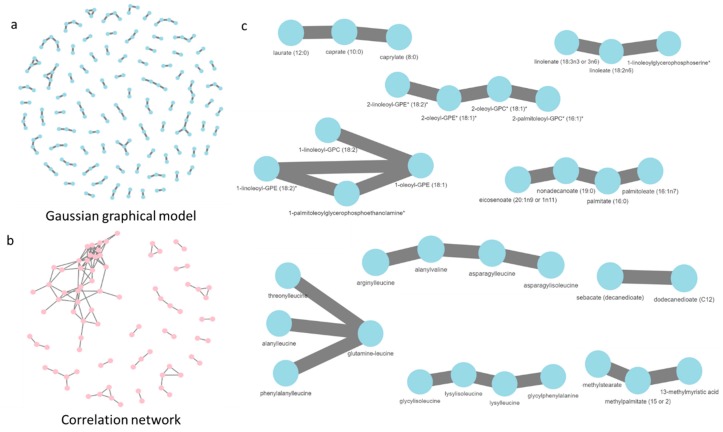
(**a**) GGM constructed using complete dataset (31 flies) showed 129 edges which included most of the edges found in the individual diet GGMs (**b**) The correlation network showed has 130 edges even with a cutoff of 0.9235, thus highlighting the inherent higher correlational structure in the metabolome (**c**) The fatty acid, phospholipid, and dipeptide subnetworks identified by GGM that are not present in correlation network (Interactive network visualization can be found in Supplemental Files: Figure_S3a_complete_ggm.html and Figure_S3b_correlation_netwrok.html).

To see how much the network topology differed among methods, we fit a GGM on the combined dataset (both ND and HFD samples, Figure 3a) and compared with the correlation network created using Pearson correlation coefficients (Figure 3b and Supplemental Table S4). The edges identified by both networks were different. The edges in the GGM were between biologically (and chemically) similar metabolites, thus doing a good job of recapitulating known metabolic pathways from quantitative data alone. For example, it successfully identified the fatty acid, phosphoplipid, and dipeptide subnetworks (Figure 3c). Edges between variables were thresholded to absolute correlation coefficients greater than 0.9235 (a very stringent cut-off of *p* < 1.36 × 10^−13^) to yield an equivalent number of edges as the GGM. In the correlation network, the edges are between a fewer number of nodes thus indicating that we are losing some relevant edges because of the high cutoff, such as in fatty acid synthesis. Moreover, the correlation network has some edges that a likely to be spurious, for example, the dipeptide subnetwork is completely interconnected in the correlation network but the GGM network is able to identify the likely dependencies within the subnetwork (Figure 3b,c). 

**Figure 4:**
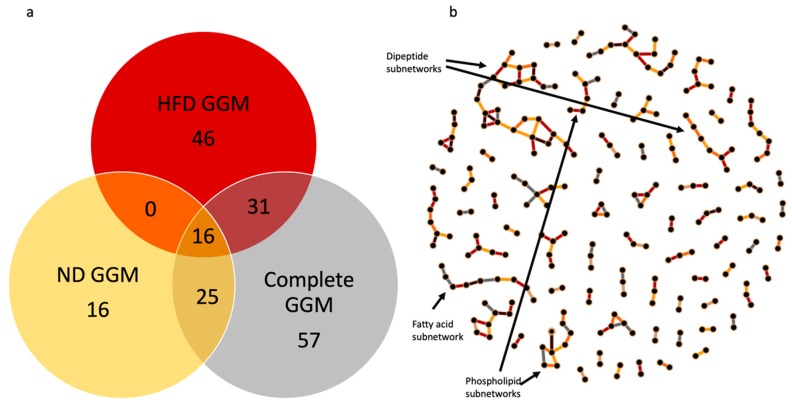
(**a**) Venn diagram showing the overlap of edges in the GGMs for the High fat diet (HFD), Normal diet (ND), and complete dataset GGMs. (**b**) The network shows the complete set of edges across all three models color coded to match the Venn diagram to specific which edges were determined from which models. (Interactive network visualization can be found in Supplemental Files: Figure_S4_all_edge_ggm.html).

To assess whether variation is edge presence/absences across datasets might be driven by overall network dependences rather than changes in the relationships between the metabolites defining the nodes of the edge, we compared the edges between HFD GGM, ND GGM, and the complete dataset GGM. The overlap in edges between the three networks is given in Figure 4 and Supplemental Table S6. To understand the shift in the relationship between two metabolites involved in an edge, we calculated Pearson’s correlation coefficient (r) for each edge in the all three models (Supplemental Table S6). We found that HFD GGM had 45 unique edges and ND GGM had 16. For the majority of the edges in these two models the correlation coefficient was higher in the respective model, compared to the other two. Further, we found that for the diet-unique edges the correlation between the node metabolites for that edge on the alternative diet was less than 0.8 for the majority of the edges (68% for HFD and 57% for the ND). This suggests that while some metabolites maintain a similar correlational relationship across diet-specific datasets, most of the conditionally dependent GGM edges unique to a given diet are indicating a genuine underlying difference between the metabolomes in the two conditions. 

We found that the edges detected in at least two of the GGMs tended to link metabolites there were well correlated across all datasets (Figure 4a, Supplemental Table S6), even if they were not linked by an edge in one of the diet specific GGMs. This suggests that these conserved edges likely capture a more stable “core” relationship among metabolites. We found 57 edges that were unique only to the complete dataset GGM. Interestingly, the correlation coefficient of majority of the edges in the complete dataset GGM model were second highest, meaning that even though there was more correlation between metabolites of these edges in HFD or ND model, still the edges only appear in the complete GGM and not individual diet GGMs.

When investigating the overlay network for the HFD, ND, and complete GGM models, we again find logical clusters (Figure 4b) and find that some of the subnetworks found in the models separately collapse in larger subnetworks when all of the edges are considered (e.g., fatty acids). Overall, the patterns of edge variation across GGM models highlights the importance of performing GGM analyses on different biologically determined subsets and combinations of the data as these will expose different aspects of the overall architecture of the organism’s metabolism.

### 2.5. The Role of Unknown Metabolites

One of the key findings in this dataset was the number of metabolites whose chemical nature and structure are unknown but that are biologically relevant, thus highlighting the limitations of the analytical techniques in metabolomics. These unknowns are possible missing links in the metabolic pathways of *Drosophila.* Random forest classification marked many of the unknowns as differentially elevated between the high-fat and normal diets. The Gaussian graphical modes associated unknowns with known network structures, thus are a good way to generate hypotheses about the class of unknown metabolites [23,25]. For example, it identified many of the unknown metabolites as part of the dipeptide subnetwork, thus they could likely belong to the dipeptide class, which is not well studied. Thus, it becomes imperative make public mass spectra from all metabolomics studies (Supplemental Table S5), which can further facilitate the identification of the unknowns and track unknowns that are shown to be biologically important across multiple studies.

## 3. Discussion

### 3.1. Effect of Diet on Lipid and Central Energy Metabolism

The higher abundance of medium chain fatty acids in HFD fed larvae is expected as they are key constituents of coconut oil [33], which was used as a primary source of fat in the HFD. Interestingly, end products of omega oxidation pathway (usually thought to be a minor pathway in the fatty acid breakdown [34]) were observed for caprate (C:10) and laurate (C:12), which are further oxidized to their monohydroxy counterparts (3-hydroxysebacate and 3-hydroxydecanoate) [35,36]. We see no elevation of the metabolites involved in the carnitine shuttle (e.g., acetylcarnitine, deoxycarnitine, propionylcarnitine, and carnitine (unconjugated)), as well as no presence of other beta-oxidation pathway metabolites [37]. Further, the increase in dicarboxylic fatty acids (decandionate and dodecandionate), in conjunction with the elevated omega oxidation products and unchanged levels of carnitine shuttle and beta oxidation products on the HFD, suggests that the excess medium chain fatty acids found in the HFD are being directed towards the omega fatty acid oxidation pathway. It is possible the dicarboxylic fatty acids are coming directly from the HFD rather than being products of the fly’s metabolism, however a complementary result to ours was identified in mice fed a coconut oil diet, wherein liver omega oxidation genes were induced to increase production of dicarboxylic fatty acids [38]. 

**Figure 5:**
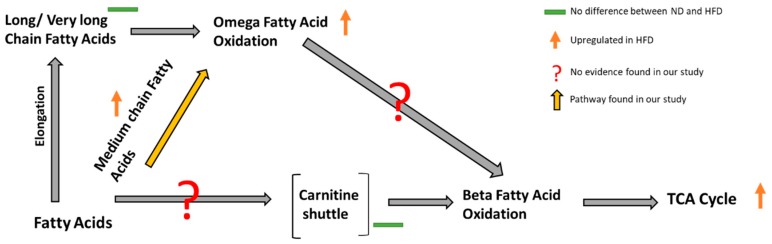
Elevation of omega fatty acid oxidation pathway and tricarboxylic acid cycle in flies fed with high-fat diet: omega fatty acid oxidation is an alternative pathway for fatty acid oxidation, which is increased in flies on HFD while there is no upregulation of more common beta fatty acid oxidation pathway.

We also see that metabolites from downstream pathways of fatty acid oxidation (glycolysis and TCA cycle) namely, glucose-6-phosphate, fructose-6-phosphate, 3-phosphoglycerate, phosphoenolpyruvate, alpha-ketoglutarate, citrate, and glutamate [39] were all upregulated in HFD larvae (Figure 5). Therefore, it needs to be further confirmed whether, products of the omega oxidation pathway go to citric acid cycle through beta-oxidation or some other pathway.

### 3.2. Effect of Triglyceride Storage on Lipid and Central Energy Metabolism

Interestingly, free fatty acids were not important variables between reaction norm phenotypes. This indicates that the higher abundance of free fatty acids correlates with diet in fly larvae irrespective of their triglyceride storage capacity. However, twelve phospholipids (Supplemental Table S3) were identified as associated with the triglyceride storage phenotype, and other studies have shown that the fatty acid composition of the phospholipids is affected by high-fat diet and exercise [40,41]. We also found eight of the phospholipids to be negatively correlated triglyceride storage levels overall, across diets and reaction norm groups (Supplemental Table S3). Aberrant levels of other phospholipid catabolic intermediates, such as glycerophosphocholine (GPC) and glycerophosphoethanolamine, are associated with a number of disease conditions, including Alzheimer’s, Down’s syndrome, and polycystic ovarian syndrome (POS) [42,43]. Subgroups of POS patients with obesity, metabolic syndrome and/or hyperandrogenism exhibited greater metabolic deviations in the lipidomic profiles of their plasma [43]. These studies have indicated that levels of these phospholipid catabolic intermediates likely have some unknown significance relating fatty acid metabolism and disease.

The class of metabolites that were important variables between phenotypic groups was the dipeptides. The elevation of dipeptides in high-fat fed larvae that stored less triglyceride could be due to elevated protein catabolism or could be due to increased dipeptide synthesis. Dipeptides are know to have bioactive effects including being potential treatments for Type-2 diabetes [44], and may be a factor in determining whether the high-fat diet of larvae induces the triglyceride storage phenotype. We believe that further exploration of dipeptides in the context of diet and microbiome may be a revealing line of inquiry in understanding the interaction between diet and genetic factors.

### 3.3. Network Analysis of Metabolites in ND and HFD Larvae

The network analysis showed many edges that were different between ND network and HFD network. Interestingly, the metabolites identified as important variables between diets by random forest are either associated with no other metabolites or at most one other metabolite in the GGMs. Also, the higher network density in HFD network compared to ND indicates greater variance among the HFD samples which could be higher biological perturbation (decanalization) in the metabolome due to the high-fat diet which has been observed for other phenotypes in flies [16]. Unfortunately, the GGM analyses alone provide little guidance on underlying mechanisms that would cause these perturbations, however GGMs remain a useful tool to develop testable hypotheses of novel pathways [25].

## 4. Materials and Methods 

### 4.1. Sample Preparation

Stocks used in this study were from the *Drosophila* Synthetic Population Resource [45,46] and were intercrossed to produce the heterozygous sampled larvae (Supplemental Table S1). Samples were then generated in the manner described in [15]. Briefly, first instar larvae were placed at densities of 50 per vial. Each vial contained 10 mL of the specific dietary treatment (normal or high-fat). The normal diet was the standard cornmeal-molasses food used in *Drosophila* stock maintenance and the high-fat treatment had the addition to the normal diet of 3% coconut oil by weight. Larvae we removed from their food treatment as late third instars, fasted for four hours on plain agar plates, then snap frozen in liquid nitrogen. Larvae from five food vials were pooled to produce the triglyceride and metabolomics samples. Using the Sigma Triglyceride assay kit as described in [15,16], triglyceride phenotypes were measured in three replicates per genotype and diet (each a pool of 10 larvae). Metabolomic samples (stored at −80 °C) consisted of 30 larvae each with one replicate per genotype and diet for a total of eight samples for each phenotype-by-diet combination. The stored samples were sent on dry ice to Metabolon Inc. for isolation and identification of metabolites. Tissue samples were extracted with methanol, using Metabolon’s standard solvent extraction method [47,48] and then samples were distributed into equal parts for Liquid Chromatography/Mass Spectrometry (LC/MS) and Gas Chromatography/Mass Spectrometry (GC/MS) analysis. Several technical replicate samples were created from a homogenous pool containing a small amount of all study samples. All samples were frozen and dried under vacuum prior to further processing. A more detailed methods description is given in [47,48]. 

### 4.2. Liquid Chromatography/Mass Spectrometry (LC/MS, LC/MS^2^) Analysis

Dried extract aliquots were resuspended in methanol: water (10:90) spiked with internal standards [47,48]. LC/MS was carried out using Waters ACQUITY Ultra Performance Liquid Chromatography (UPLC) and a Thermo-Finnigan Linear Trap Quadrupole (LTQ) mass spectrometer, consisting of an electron spray ionization (ESI) source and linear ion-trap (LIT) mass analyzer. For LC/MS^2^ analysis, additional Fourier transform ion cyclotron resonance (FT-ICR) mass spectrometer was used at the backend if accurate mass was needed for a compound ID. The typical mass error was less than 5 ppm (parts per million). More detailed method employed can be found at [47,48,49]. Briefly, the sample extract was split into two aliquots, one was analyzed using acidic positive ion optimized conditions and other using basic negative ion optimized conditions on separate dedicated columns. The acidic extracts were gradient eluted using water and methanol containing 0.1% formic acid, and the basic extracts were gradient eluted using water and methanol containing 6.5 mM ammonium bicarbonate. The MS analysis alternated between MS and data-dependent MS^2^ scans using dynamic exclusion. All samples were run in a single batch in a randomized order.

### 4.3. Gas Chromatography/Mass Spectrometry (GC/MS) Analysis

For GC/MS analysis, aliquots of the dried extract were derivatized under dried nitrogen at at 60 °C for 1 h with a solution containing N,O-bis[trimethylsilyl]trifluoroacetamide (BSTFA) and acetonitrile (ACN):dichloromethane (DCM):cyclohexane (5:4:1, v/v/v) with 5% triethanolamine (TEA) in equal parts, as well as internal standards [48]. The GC column used was 5% phenyl and the temperature ramp was from 40 °C to 300 °C over a 16-min period. The analysis was performed using Thermo-Finnigan Trace Dual Stage Quadrupole (DSQ) fast-scanning mass spectrometer using electron impact ionization. All samples were run in a single batch in randomized order.

### 4.4. Data Quality and Metabolite Identification

Instrument variability was determined to be 4% by calculating the median relative standard deviations (RSD) for the internal standards that were added to each sample prior to injection into the mass spectrometers. Total process variability was determined to be 11% by calculating the median RSD for all endogenous metabolites present in 100% of samples, which are technical replicates of pooled samples.

The raw mass spec data files were loaded into an inhouse relational database without BLOB manipulation. Peaks were identified using Metabolon’s proprietary peak integration software. Metabolites were identified based on combination of mass and retention time that matched with Metabolon’s in-house library consisting of purified standards and recurrent unknown entities [50]. Quality check and curation were performed by Metabolon using proprietary methods which included confirmation of consistency of peak identification among the various samples and standards. Library matches for each compound for each sample were assessed to ensure accurate identification of metabolites and to remove system artifacts, mis-assignments, and background noise.

### 4.5. Data Pre-Processing and Analysis

Compound measurements were normalized based on an internal protein standard. Any missing values were imputed to the minimum detected value in the dataset for that metabolite and log transformed. We removed one sample that was fed a high-fat diet from the dataset as the relative concentrations values were abnormally high. The final dataset consisted of 31 samples, 15 of which were fed high-fat diet and 16 were fed normal diet. For reaction norm phenotype analysis, we had 12 genotype-by diet-combinations that represented the reaction norm that stored 25% more triglyceride on a high-fat diet than on a normal diet (*N < HF*), 9 that represented the reaction norm that stored 25% more triglyceride on normal diet than a high-fat diet (*N > HF*), and 10 representing the reaction norm that showed no significant change in triglyceride storage across diets (less than 25% change, supplemental Table S1 and supplemental Figure S1a).

We found that PCA analysis of the complete data set did not differentiate the samples by diet or reaction norm (Supplemental Figure S1b). Thus, we used random forest analysis to identify key metabolite variables between the high-fat and normal diets, as well as between reaction norm phenotypes (N > HF and N < HF). Random forest uses an ensemble of decision trees (n) using a pre-specified number of randomly selected variables (m) from a total number of variables (M) to identify variables that best classify the data into different groups [20]. The analysis was done using the randomForest package [version 4.6] in R [51], with 1000 trees and the predefined variable cut-off (m) of 18, as it gave the lowest out-of-bag error rate across five different values of m (5, 18, 100, 250 and 350) and is also close to the square root of the total number of variables (metabolites), which is the recommended value by Breiman [20]. After all of the 1000 trees have been grown, the biological samples that did not participate in the training of trees are used as a test set to get test error rate for each tree. The out-of-bag (OOB) error rate is then calculated as a test error averaged over all 1000 trees grown. Hence, the lower the OOB error rate, the better the classification. The variable importance score is determined by the ranking each variable (metabolite) gets as an “important” classifier for a particular condition. The importance of the variable is determined by the decrease in accuracy in predicting the correct class of out-of-bag samples when the variable is removed from the “bag”. The more the accuracy decreases, the more important the variable [20,21,51]. The top 10 important variables were subsequently subjected to hierarchical clustering [52] using Ward’s algorithm [53,54]. A PCA analysis of the top 10 important metabolites for diet did separate the samples nicely as would be expected once the subset of informative metabolites was determined (Supplemental Figure S1c).

ANOVA was also used to identify metabolites that significant dietary, reaction norm, and diet-by-reaction norm interactions. Post hoc tests using a Student’s *t*-test were used to identify the significant contrasts between reaction norm groups. Metabolite concentrations were also regressed against triglyceride concentrations to identify metabolites that correlated with triglyceride levels across treatments. 

Gaussian graphical models (GGMs) were constructed for the normal diet, high-fat diet, and combined samples using the GGMselect package [version 0.1-12] in R [55]. The GGMselect algorithm is suitable for handling high dimensional data where the sample size (n) is less than the number of variables (p) and uses a two-step procedure: 1—build a data-driven family of graphs using nodewise regression with the Lasso as in [56], 2—select the graph with the best penalty score [55]. To identify a sparse model that minimizes false positives, the GGMselect penalty function relies on two parameters, dmax—the maximum degree for a node, and K—the scalar tuning parameter whose value should be between two and five (a higher K value means stricter penalty function) [55]. Increasing the value of dmax tends to increase the number of edges, and increasing the value of K decreases the number of edges in the network. After generating networks with different values of K (between two and five) and dmax (between one and ten), we found that with even at lower penalty (K = 2) the algorithm plateaued at 129 edges while dmax was set between four and ten. This suggests that even with minimum constraints the GGMselect algorithm selects a sparse network. Hence we set K = 2 and dmax = 5 as our default parameters for GGM network generation. To quantify the difference between the HFD GGM and ND GGM, we calculated the edge symmetric difference (esd) [32] between the networks, which is given by:

$$esd\left(HF,N\right)=\;\frac{\left|E\left(HF\right)\backslash E\left(N\right)\right|+\left|E\left(N\right)\backslash E\left(HF\right)\right|}{\left|E\left(HF\right)\left|+\right|E\left(N\right)\right|}$$

where |E(HF)\E(N)|  is equal to the number of edges belonging to the HFD network but not to the ND network and |E(N)\E(HF)| is equal to the number of edges belonging to the ND network but not to the HFD network. |E(HF)| is the total number of edges in the HFD network, and |E(N)| is the total number of edges in the ND network. Thus, this esd value represents the proportion of edges that are not shared between the two networks and thus is a value between zero and one; values closer to zero represents similar networks while values closer to one represent dissimilar networks. 

We also constructed a correlation network using Pearson’s correlation coefficients and compared with the GGM network to see if GGM performed better at identifying edges that represented relevant biological relationships. A correlation of 0.9235 was used a cut-off for the presence of an edge to produce the correlation network with a comparable number of edges to what was observed in the GGM. 

## 5. Conclusions

Although there have been attempts to map the *Drosophila* metabolites in different tissues [57] and conditions [15,58,59,60,61,62,63,64] and predict possible metabolic pathways based on the annotated genome [65], a lack of a comprehensive *Drosophila* metabolome and metabolic pathways database based on metabolomics data has hindered the progress of untargeted metabolomics studies in *Drosophila*. The current study supports the potential of untargeted studies and its utilization as a dynamic pathway analysis tool, even with limited prior knowledge. Considering the cost of metabolomics studies, metabolomics datasets are often smaller and therefore at risk of being overfit in analyses of this high dimensional data [66]. In this study, we have successfully employed random forest as a conservative approach for identification of key metabolites from a large repertoire, as well as Gaussian graphical models with the Lasso algorithm to identify how metabolite network dynamics change between different conditions. The study identified several key effects of the high-fat diet on lipid and central energy metabolism, identifying numerous metabolites previously unreported to be a part of any standard metabolic pathway in the KEGG database for *Drosophila.* For example, extended analysis of important omega fatty acid oxidation intermediates indicates possible alternative pathway(s) that should be further investigated to broaden our understanding of fatty acid metabolism. Further, our results, observing no elevation of the metabolites involved in carnitine shuttle, supported the previous reports [34,67] of medium chain fatty acids being a directed towards the omega fatty acid oxidation pathway in disease conditions. Highlighting the role of this alternative fatty acid oxidation pathway provides new insight into the role of diet as a contributor to metabolic phenotypes. The pattern of elevated dipeptides in larvae eating a high-fat diet while storing less triglyceride suggests these groups of metabolites as potentially important mediators or indicators of metabolic health in the face of a perturbing diet. The GGM networks differed greatly between two diets highlighting different metabolic pathway perturbations. Overall, the approach used in this study will aid the process of creating a global *Drosophila* metabolome model and can be also used to develop metabolic hypotheses in biological systems less well-characterized than *Drosophila.*

## Supplementary Materials

The following are available online at http://www.mdpi.com/2218-1989/9/1/5/s1, Figures: Figure_S1a.png, Figure_S1b.png, Figure_S1c.png, Figure_S2a_normal_ggm.html, Figure_S2b_highfat_ggm.html, Figure_S2c_combined_ggm.html, Figure_S3a_complete_ggm_html, Figure_S3b_correlation_network.html, Figure_S4_all_edge_ggm.html Table: Supplementary_Tables.xlsx.
