# Supplementary material for: Random Forest Analysis of Untargeted Metabolomics Data Suggests Increased Use of Omega Fatty Acid Oxidation Pathway in Drosophila Melanogaster Larvae Fed a Medium Chain Fatty Acid Rich High-Fat Diet
Source: Metabolites. 2018 Dec 31;9(1):5. doi: 10.3390/metabo9010005 (PMC6359074; doi:10.3390/metabo9010005)
Supplement: Supplementary file 1 [file metabolites-09-00005-s001.zip › supplementary_files/Figure_S2a_normal_ggm.html]

visNetwork
