# Supplementary figures and images for: Random Forest Analysis of Untargeted Metabolomics Data Suggests Increased Use of Omega Fatty Acid Oxidation Pathway in Drosophila Melanogaster Larvae Fed a Medium Chain Fatty Acid Rich High-Fat Diet
Source: Metabolites. 2018 Dec 31;9(1):5. doi: 10.3390/metabo9010005 (PMC6359074; doi:10.3390/metabo9010005)

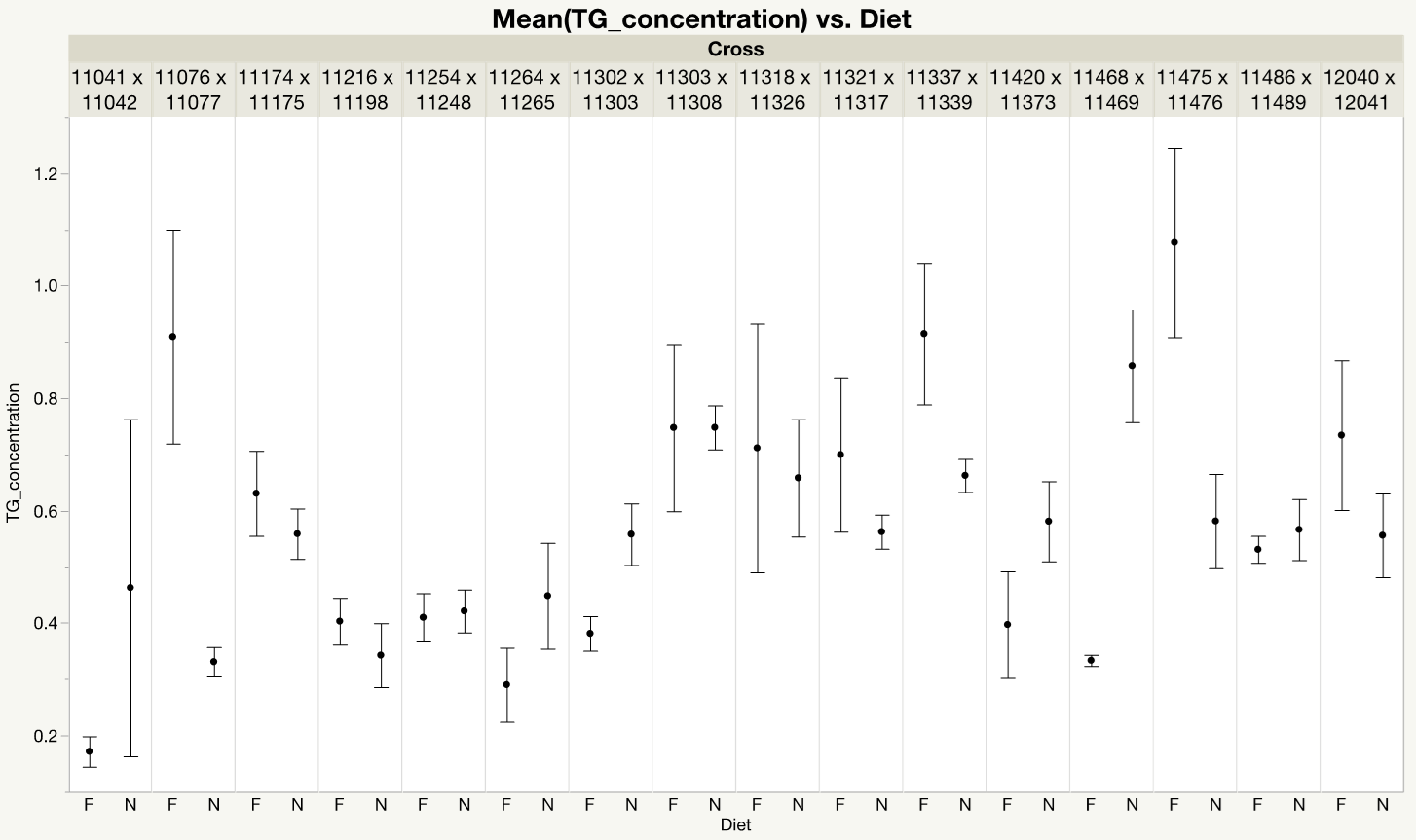

Supplement: Supplementary file 1 [file metabolites-09-00005-s001.zip › supplementary_files/Figure_S1a.png]

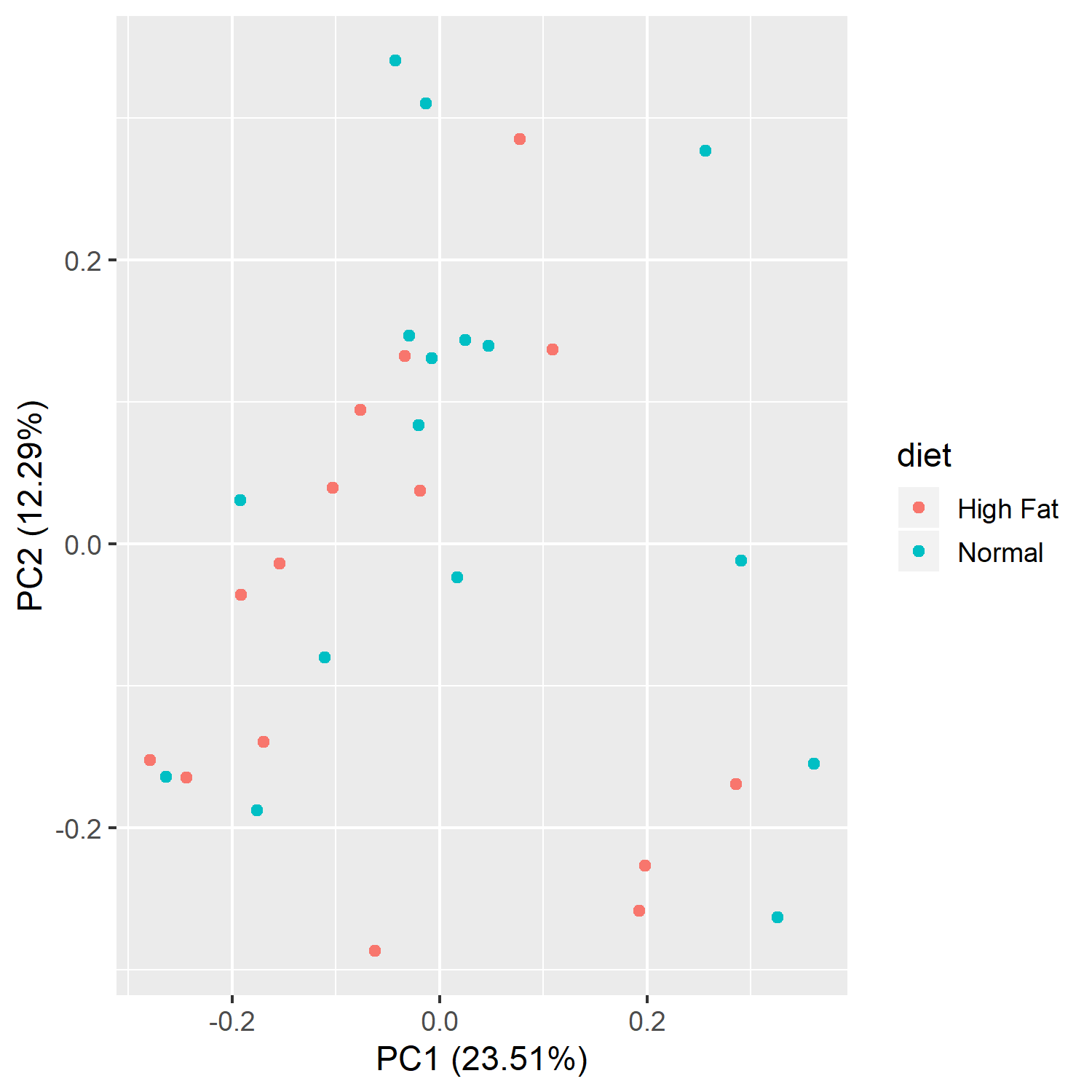

Supplement: Supplementary file 1 [file metabolites-09-00005-s001.zip › supplementary_files/Figure_S1b.png]

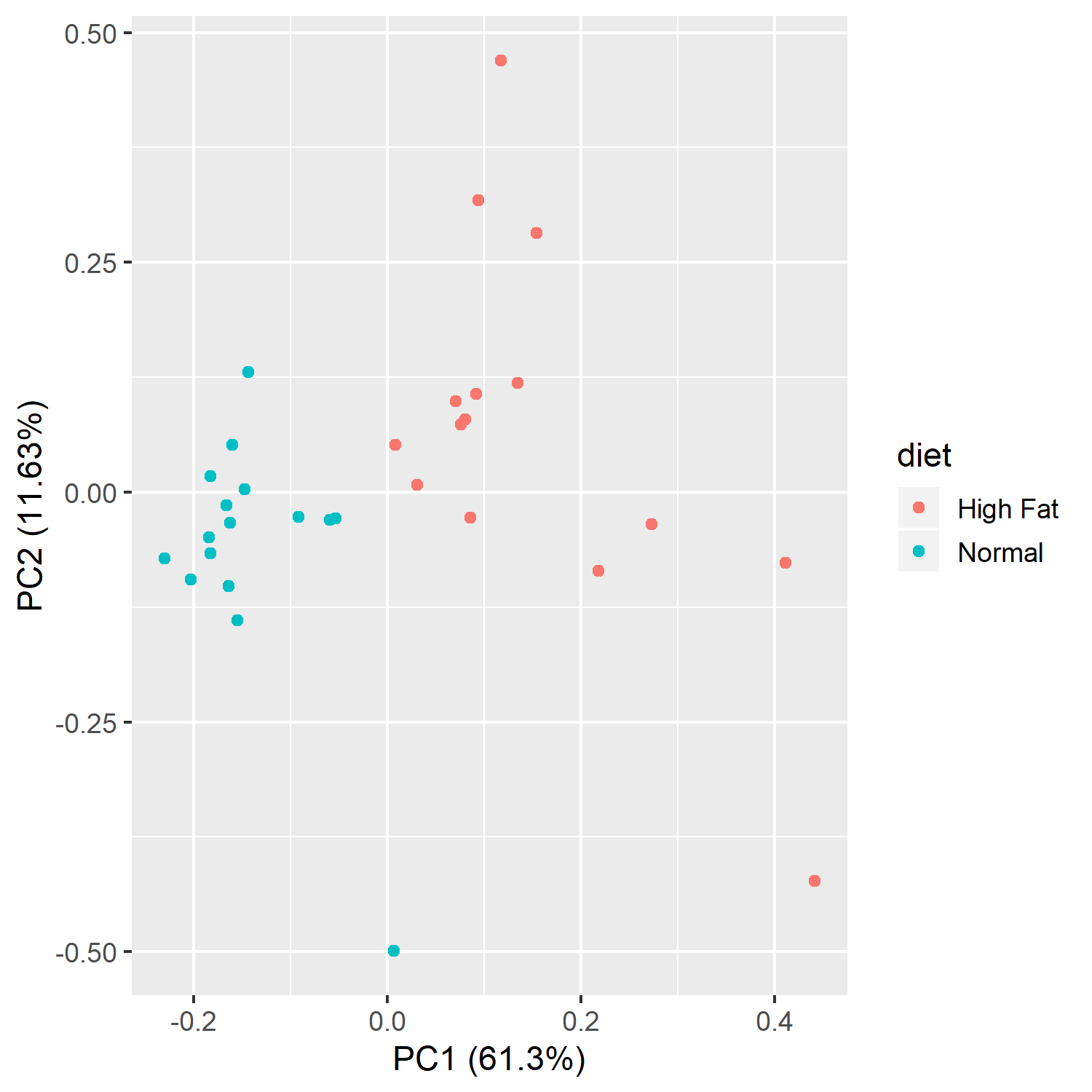

Supplement: Supplementary file 1 [file metabolites-09-00005-s001.zip › supplementary_files/Figure_S1c.png]

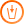

Supplement: Supplementary file 1 [file metabolites-09-00005-s001.zip › supplementary_files/Figure_S2a_normal_ggm_files/vis-4.20.1/img/network/acceptDeleteIcon.png]

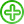

Supplement: Supplementary file 1 [file metabolites-09-00005-s001.zip › supplementary_files/Figure_S2a_normal_ggm_files/vis-4.20.1/img/network/addNodeIcon.png]

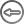

Supplement: Supplementary file 1 [file metabolites-09-00005-s001.zip › supplementary_files/Figure_S2a_normal_ggm_files/vis-4.20.1/img/network/backIcon.png]

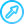

Supplement: Supplementary file 1 [file metabolites-09-00005-s001.zip › supplementary_files/Figure_S2a_normal_ggm_files/vis-4.20.1/img/network/connectIcon.png]

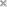

Supplement: Supplementary file 1 [file metabolites-09-00005-s001.zip › supplementary_files/Figure_S2a_normal_ggm_files/vis-4.20.1/img/network/cross.png]

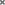

Supplement: Supplementary file 1 [file metabolites-09-00005-s001.zip › supplementary_files/Figure_S2a_normal_ggm_files/vis-4.20.1/img/network/cross2.png]

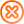

Supplement: Supplementary file 1 [file metabolites-09-00005-s001.zip › supplementary_files/Figure_S2a_normal_ggm_files/vis-4.20.1/img/network/deleteIcon.png]

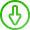

Supplement: Supplementary file 1 [file metabolites-09-00005-s001.zip › supplementary_files/Figure_S2a_normal_ggm_files/vis-4.20.1/img/network/downArrow.png]

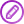

Supplement: Supplementary file 1 [file metabolites-09-00005-s001.zip › supplementary_files/Figure_S2a_normal_ggm_files/vis-4.20.1/img/network/editIcon.png]

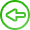

Supplement: Supplementary file 1 [file metabolites-09-00005-s001.zip › supplementary_files/Figure_S2a_normal_ggm_files/vis-4.20.1/img/network/leftArrow.png]

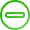

Supplement: Supplementary file 1 [file metabolites-09-00005-s001.zip › supplementary_files/Figure_S2a_normal_ggm_files/vis-4.20.1/img/network/minus.png]

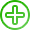

Supplement: Supplementary file 1 [file metabolites-09-00005-s001.zip › supplementary_files/Figure_S2a_normal_ggm_files/vis-4.20.1/img/network/plus.png]

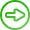

Supplement: Supplementary file 1 [file metabolites-09-00005-s001.zip › supplementary_files/Figure_S2a_normal_ggm_files/vis-4.20.1/img/network/rightArrow.png]

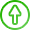

Supplement: Supplementary file 1 [file metabolites-09-00005-s001.zip › supplementary_files/Figure_S2a_normal_ggm_files/vis-4.20.1/img/network/upArrow.png]

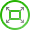

Supplement: Supplementary file 1 [file metabolites-09-00005-s001.zip › supplementary_files/Figure_S2a_normal_ggm_files/vis-4.20.1/img/network/zoomExtends.png]
